# Few-View Prereconstruction Guided Tube Current Modulation Strategy Based on the Signal-to-Noise Ratio of the Sinogram

**DOI:** 10.1155/2015/906452

**Published:** 2015-05-18

**Authors:** Ming Chang, Yongshun Xiao, Zhiqiang Chen

**Affiliations:** ^1^Department of Engineering Physics, Tsinghua University, Beijing 100084, China; ^2^Key Laboratory of Particle & Radiation Imaging, Tsinghua University, Ministry of Education, Beijing 100084, China

## Abstract

The radiation dose reduction without sacrificing the image quality as an important issue has raised the attention of CT manufacturers and different automatic exposure control (AEC) strategies have been adopted in their products. In this paper, we focus on the strategy of tube current modulation. It is deduced based on the signal-to-noise (SNR) of the sinogram. The main idea behind the proposed modulation strategy is to keep the SNR of the sinogram proximately invariable using the few-view reconstruction as a good reference because it directly affects the noise level of the reconstructions. The numerical experiment results demonstrate that, compared with constant tube current, the noise distribution is more uniform and the SNR and CNR of the reconstruction are better when the proposed strategy is applied. Furthermore it has the potential to distinguish the low-contrast target and to reduce the radiation dose.

## 1. Introduction

X-ray CT has played an important role in primary diagnostic imaging and radiotherapy since its introduction in 1973. It is estimated that 67 million CT examinations were performed in 2006 in the USA while the number was about 3 million in 1980 [1]. With the increasing utilization of CT, the radiation dose and corresponding potential risks associated with CT scanning raise the ongoing concern to both the patients and CT manufactures. According to the as low as reasonably achievable principle (ALARA), the radiation dose reduction is an important issue in clinical routine, methodology research, and system development.

Various factors, including system scanning parameters, the difference of the patients, and the requirement for the following diagnoses, have influences on the CT imaging dose. The CT dose index (CTDI) is a measure of the absorbed dose to a standard plastic phantom, which is commonly used as the CT dose metric [2, 3]. It is affected by the scanning mode, exposure time, tube current, tube potential, field of measurement, and beam shape filtering [2, 4–6]. Generally it is directly proportional to the tube current and the exposure time. It is approximately proportional to the square of the percentage change in tube potential. These parameters can be adjusted flexibly according to the specific imaging task. Therefore the adjustment strategy of these parameters according to the specific applications is the key to achieve a lower CTDI in practical CT examinations.

The automatic exposure control (AEC) technology is to automatically adapt the tube current or tube potential according to patient's attenuation to achieve a specified image quality. In the currently used commercial CT system, the AEC technologies based on different strategies are adopted [7]. Automatic tube potential selection is to choose the tube potential according to the patient size or image task in order to achieve the desired image quality with a lower CTDI. Angular and longitudinal current modulation are to adjust the tube current according to the patient size, shape, and attenuation changes at different projection views [4]. Different vendors provide strategies based on slightly different principles [8]. GE adopts AutomA and SmartmA theory based on the noise index (NI) of the reference image, which is used to control the average image noise level [9, 10]. It is possible to achieve clinically acceptable images at the lowest radiation dose to patients using the optimal NI selection and model-based iterative reconstruction (MBIR) method [11]. Philips offers the DoseRight technology and uses a reference image concept. Quality reference mAs and reference standard deviation are selected by Siemens and Toshiba, respectively [7]. However the adjustment based on the online feedback can be only used for helical scanning [12] and the strategy based on the predictive calculation or sinusoidal-type function may be far apart from the real situation. To our knowledge, most currently used strategies are designed based on the guideline of image quality and adaptive statistical iterative reconstruction (ASIR) or MBIR methods are necessary [11, 13]. As we know, the noise level of the sinogram directly affects the CT image quality no matter what reconstruction method is used. Therefore the SNR of the sinogram is considered when the proposed method is designed.

In this study, we focus on the tube current modulation strategy in the CT scanning. In order to achieve a better image quality of the reconstruction, we propose the few-view prereconstruction guided tube current modulation strategy. It is established based on the analysis of the noise in the sinogram, which directly affects the final image quality. The main idea behind the proposed strategy is to keep the SNR of the sinogram proximately invariable at different angle views using the few-view prereconstruction as a good reference for the adjustment of the tube current. The rest of the paper is organized as follows. In the next section, the proposed strategy derived from the noise analysis is introduced. A specific workflow using the proposed strategy to enhance the image quality of the reconstructed images is also presented in this section. In the third section, numerical experiments are carried out and qualitative and quantitative results are shown correspondingly. In the end, the conclusions are made for this work.

## 2. Materials and Methods

### 2.1. Tube Current Modulation Strategy Based on the SNR of the Sinogram

According to the Lambert-Beer law, the ideal attenuated X-ray photon *I* is expressed as

(1) $$\begin{array}{c} I=I_{0}\exp\left(-p\right), \end{array}$$

where *I*_0_ is the initial photon and *p* is the integration of the linear attenuation coefficients along the X-ray path.

Generally, the quantum noise and the system electronic noise exist in the practical CT measurements. The system electronic noise should be taken into account in the case of low-dose CT [14]. However, in this work, only the Poisson distributed quantum noise is considered in the following analysis. With the Poisson statistics, the practical measurement *I*_*m*_ is presented in

(2) $$\begin{array}{c} I_{m}=\text{Poisson}\left(I\right)\approx I+\sqrt{I}x\left(0,1\right), \end{array}$$

where *x*(0,1) represent the random value, which is satisfied with the standard normal distribution.

Before CT reconstruction, the measurements are converted into the sinogram *p*_*m*_ by the negative logarithm operation:

(3) $$\begin{array}{c} p_{m}=-\log\left(\frac{I_{m}}{I_{0}}\right)=p-\log\left[1+\frac{1}{\sqrt{I}}x\left(0,1\right)\right]. \end{array}$$

Then the noise of the sinogram Δ*p* is presented as follows:

(4) $$\begin{array}{c} \Delta p=\left|p_{m}-p\right|\approx \frac{1}{\sqrt{I}}x\left(0,1\right). \end{array}$$

The SNR of the sinogram is defined as the ratio of the mean value to the standard variance, which should be expressed as

(5) $$\begin{array}{c} \text{SN}{\text{R}}_{p}=\frac{p}{\sigma_{\Delta p}}=\frac{p\sqrt{I_{0}}}{\exp\left(p/2\right)}=k. \end{array}$$

If the *p* is a variable in 5, the SNR_*p*_ achieves a global minimization by making *p* = 2.0. It can be used for the tube potential modulation. However, we only focus on the current modulation strategy in this work. The *p* is no longer a variable after the tube potential is set for a specific imaging task. To achieve a reconstructed image with uniformly distributed noise, the SNR_*p*_ should be a constant *k* or at least should not be changed significantly. This can be realized by adjusting the initial intensity *I*_0_ according to the attenuation coefficient *p*. Based on such principle, the tube current modulation strategy can be deduced from 5:

(6) $$\begin{array}{c} I_{0}=\frac{k^{2}\exp\left(p\right)}{p^{2}}. \end{array}$$

Equation 6 provides the basis of tube current modulation. For a fixed total photon count *I*_total_, the allocation of the photon count for each view should be done based on

(7) $$\begin{array}{c} I_{0i}=\frac{w_{i}}{\sum_{i}^{}w_{i}}I_{\text{total}},\;\text{where}\;w_{i}=\frac{\exp\left(p_{i}\right)}{{p_{i}}^{2}}. \end{array}$$

The subscript *i* indicates the index of the view angles. By 7, on one hand, the number of required initial photons increases with the growth of the *p*_*i*_ when the *p*_*i*_ is greater than 2.0. On the other hand, it also increases with the decrease of *p*_*i*_ when *p*_*i*_ is less than 2.0. The least amount of initial photos is needed by *p*_*i*_ = 2.0.

### 2.2. Strategy Implementation

However, there are still two problems to be addressed when the strategy is implemented. The first one is how to determine the weight factor *w*_*i*_ for each view in 7. In the practical CT scanning, the attenuations of different detector bins are generally not the same at a certain view angle and it is difficult to adjust the tube current for each detector bin. As aforementioned, the SNR of the sinogram is expected to be constant or invariable approximately. Therefore the difference of the sinogram's SNR from the desired one within a certain projection view should be minimized:

(8) pi=argminpiΔSNRipi,where  ΔSNRi(pi)=∑jSNRqij−SNRpi2.

The *q*_*ij*_ is the attenuation coefficient obtained by *j*th detector bin at *i*th projection view. The *p*_*i*_ determines the SNR_*p*_*i*__ at the *i*th projection view. Then the weight factor *w*_*i*_ in 7 can be expressed as

(9) $$\begin{array}{c} w_{i}={\left[\frac{M}{\sum_{j=1}^{M}q_{ij}\exp\left(-q_{ij}/2\right)}\right]}^{2}. \end{array}$$

The *M* indicates the total number of the detector bins. It should be noted that the weight factor in 9 is a compromise of the high attenuation projection and the low attenuation projections in terms of the SNR. In fact, the tube current is too low for the projections of high attenuation and relatively high for the projections of low attenuation. In order to suppress the strips artifacts caused by the poor SNR of high attenuation projection, the projections *q*_*ij*_ used to calculate the weight factor *w*_*i*_ in 9 should be above a certain threshold. The threshold *T*_*i*_ is projection view related and it is determined by the median of *q*_*ij*_  (*j* = 1,2,…, *M*) within the corresponding view in the following experiments. It should be noted that it is an empirical parameter and it may not be the optimal choice. But it works well in our study.

The second problem is to get the sinogram *q*_*ij*_ as a reference for the tube current modulation. However it is impossible to achieve such a sinogram before completing the scanning. In practical CT scanning, patient sizes, shapes, and compositions may differ from the assumption, which has the negative influence on the tube current modulation. In this study, a few-view prereconstruction image is used to acquire the sinogram for the determination of the weight factors in the proposed strategy. Recently the compressed sensing based reconstruction methods make it possible to achieve the reconstructed image of the acceptable image quality using few-view projection data [15–18]. The few-view reweighted sparsity hunting (FRESH) method, which is demonstrated to have good performance in the case of few-view tomography, is adopted to complete the task. You can refer to [17] for the details about the method. Then the forward projection operation is done with the few-view prereconstruction and the simulating sinograms provide a good guide for the specific modulation plan in the following routine scanning.

**Figure 1:**
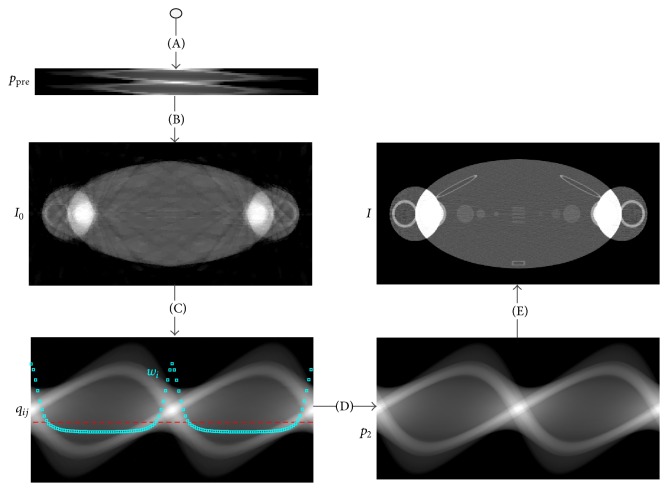
The complete work flow of the proposed strategy.

The complete work flow of the proposed strategy is shown in Figure 1. (A) few-view scanning to get the sinogram *p*_pre_; (B) carrying out prereconstruction *I*_0_ by FRESH method using *p*_pre_; (C1) estimating the complete sinogram *q*_*ij*_ by forward projection of *I*_0_; (C2) calculating the weight factor *w*_*i*_, according to 9 using *q*_*ij*_; (D) routine scanning with the strategy based on the weight factor *w*_*i*_ to get the sinogram *p*_2_; (E) carrying out final reconstruction *I* with *p*_2_.

## 3. Results and Discussion

**Figure 2:**
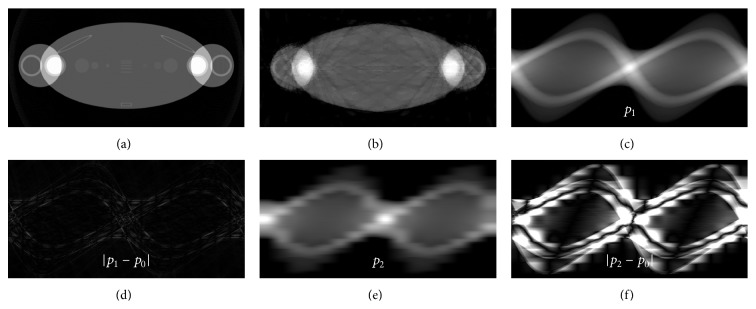
The thorax phantom and the sinogram used for the determination of the weight factors. (a) The thorax phantom; (b) the prereconstruction using 16-view projections; (c) the estimated sinogram using the prereconstruction *p*_1_; (d) the difference image |*p*_1_ − *p*_0_|; (e) the estimated sinogram by interpolation *p*_2_; (f) the difference image |*p*_2_ − *p*_0_|. The display windows are set to [0, 0.3] for (a) and (b), [0, 7] for (c) and (e), and [0, 1] for (d) and (f).

In the first experiment, as shown in Figure 2(a), the thorax phantom was used to demonstrate the feasibility and effectiveness of the proposed strategy. In the phantom, there were arms, clavicle, humerus, and shoulder blades of high attenuations. Several low-contrast disks and the line-pair were placed in the center of the phantom. They were used to test the distinguish ability of the soft tissue. The initial photon intensity was set to 1.0 × 10^5^ for each view. The geometrical configuration parameters in the simulations are listed in Table 1.

**Figure 3:**
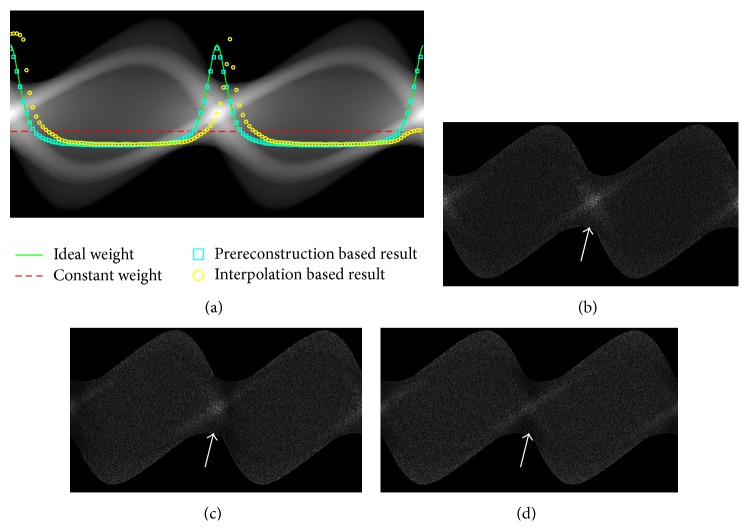
The weight factors based on different references for tube current modulation and the corresponding sinogram acquired based on such strategy. (a) The weight factor based on different strategies; (b)–(d) the reciprocal of the sinogram's SNR using constant weight factor and interpolation based and prereconstruction based weight factors, respectively. The display windows are set to [0, 7] for (a) and [0.00, 0.035] for (b)–(d).

To have a clear analysis of the proposed strategy, the intermediate result is presented in the first numerical simulations. Firstly, a prereconstruction by the FRESH method was done using the uniformly distributed 16-view projections and the result is shown in Figure 2(b). It was used to estimate the sinogram for the current modulation strategy in the following routine scanning. As the results shown in Figures 2(c) and 2(d), the estimated sinogram *p*_1_ using the prereconstruction image as a good reference was very close to the ideal one *p*_0_. In contrast, there were significant errors in the direct interpolations' result *p*_2_ using the few-view projections, which eventually leads to an inappropriate current modulation. To give a clear illustration of their influence on the proposed strategy, the comparison of the determined weight factors based on different references was plotted in Figure 3(a). It can be found that the prereconstruction based result was consistent with the ideal one. More photons were distributed at the projection views where the attenuation is relatively high and slightly less photons were used in the other view angles. It makes it possible to achieve a more uniformly distributed SNR of the sinogram. However the interpolation based result did not match well at some view angles due to the errors of the interpolation based sinogram. Figures 3(b)–3(d) show the reciprocal of the sinogram's SNR using different weight factors for the tube current modulations. As the white arrows indicated, compared to the other strategy, the SNR was enhanced at the projection views where the attenuation is relatively high by the proposed strategy, which yields a more uniformly distributed SNR of the sinogram.

**Figure 4:**
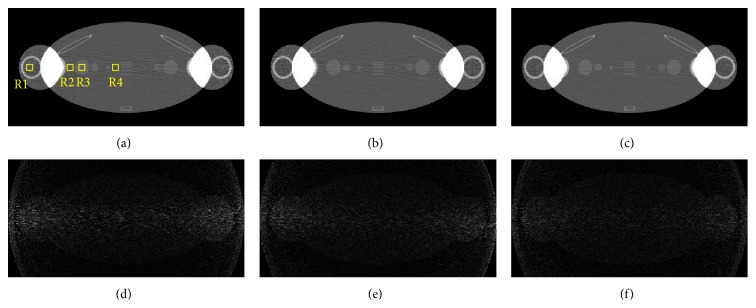
The comparisons of the reconstructions using different strategies with the thorax phantom. Reconstructions using a constant current and interpolation based strategy and the proposed strategy are shown in (a)–(c). The corresponding difference images from the phantom are presented in (d)–(f), respectively. The display windows are set to [0.05, 0.20] for (a)–(c) and [0.00, 0.05] for (d)–(f).

Then the routine scanning with the proposed strategy was done. It was compared with the scanning using a constant tube current. However, it should be noted that the total photon of each whole scanning is the same. The comparisons in the following experiments were all based on such premise. The results in Figure 4 show that the noise distribution in the final reconstruction was more uniform using the proposed strategy than using a constant tube current or the interpolation based method. As Figure 4(a) has shown, the result of constant weight is corrupted with severe strips artifacts. Although some improvements on image quality have been achieved in the interpolation based result Figure 4(b), there were some obvious strips artifacts due to the imperfect current modulation. By contrast, the low-contrast disk and the line-pair in Figure 4(c) could be easily distinguished. To make a quantitative analysis of the image quality, the SNR and CNR of the 10 × 10 pixel^2^ region of interest (ROI) were calculated. The pixel size is 0.0879 cm and the dimensions of the ROI are 0.879 ∗ 0.879 cm^2^. The definitions are given in 10 and 11. The ROIs were labeled as R1–R4 in Figure 4(a) and they were used to calculate the SNR. The CNR was estimated based on R2 and R3. As listed in Table 2, the SNR and CNR along the lateral direction have been greatly improved due to enhancements of the sinogram's SNR in these directions:

(10) $$\begin{array}{c} \text{SNR}=\frac{\mu }{\delta }, \end{array}$$

where the *μ* and *δ* are the mean and the standard deviation of the ROI. Consider

(11) $$\begin{array}{c} \text{CNR}=\frac{\left|\mu_{1}-\mu_{2}\right|}{\delta_{0}}, \end{array}$$

where *μ*_1_ and *μ*_2_ are the mean of R1 and R2 and *δ*_0_ is the standard deviation of the pure image noise.

**Figure 5:**
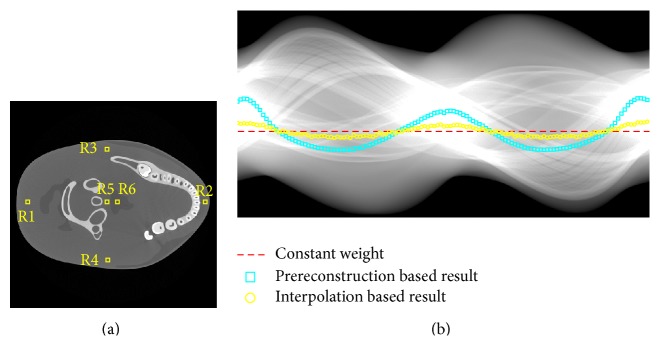
(a) The dental phantom and (b) the sinogram obtained using the proposed strategy with the weight factor based on the prereconstruction plotted on it. The display window is set to [0.0, 0.5] and [0, 7], respectively.

In the second experiment, a more complicated dental phantom, as shown in Figure 5(a), was used to further demonstrate the effectiveness of the proposed strategy for the practical applications. It was done on the CT simulation platform developed by our group [19]. The phantom was designed based on a real CT image using B-spline curves to approximate the edges of different compositions. The X-ray spectrum of 160 kV used in this experiment was simulated by Monte Carlo method. The corresponding attenuation coefficients of various tissues, including the adipose, dentin, brain, and enamel, at different X-ray energy were obtained from the web of the National Institute of Standards and Technology (NIST). The material of the detectors was CsI and the response to different X-ray energy was also considered in our simulations. The geometrical configuration parameters in the simulations were similar to the previous set.  Figure 5(b) shows the sinogram obtained by the proposed strategy. The weight factor in such strategy is determined by the prereconstruction. Similar to the results in the first experiment, the distribution of the initial photon intensity is more reasonable in terms of the sinogram's SNR. Compared with the result in the first experiment, the amplitude of the weight curve becomes small because the differences of the sinogram at different views are not as significant as they were in the first experiment.

**Figure 6:**
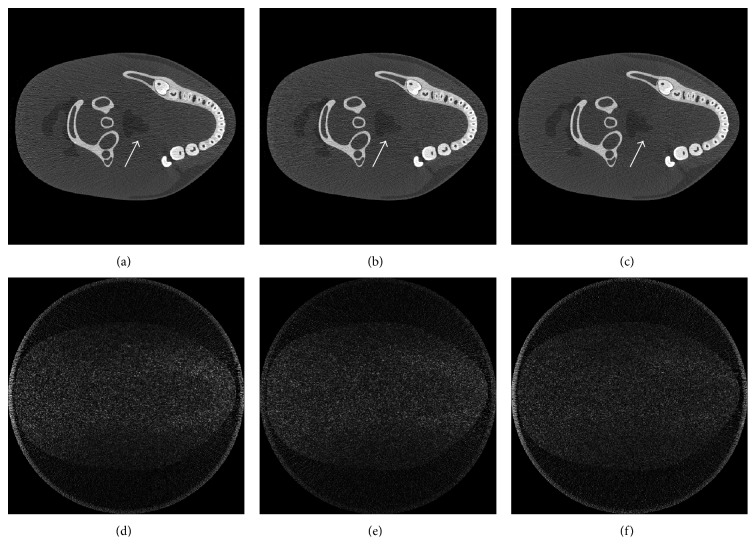
The comparisons of the reconstructions using different strategies with the dental phantom. Reconstructions using a constant current and interpolation based strategy and the proposed strategy were shown in (a)–(c). The corresponding difference images from the phantom were presented in (d)–(f), respectively. The display windows are set to [0.1, 0.5] for (a)–(c) and [0.00, 0.15] for (d)–(f).

The results using the proposed method as well as other strategies are shown in Figure 6. The distribution of the image noise in our result was more uniform, especially along the direction where the X-ray was attenuated seriously. As the arrow indicated, the edge of the low-contrast structure was easily distinguished in Figure 6(c) while it was corrupted by the noises and artifacts in Figures 6(a) and 6(b). The SNR and CNR were also calculated for the quantitative analysis in this experiment. The ROIs were labeled in Figure 5(a). As the results listed in Table 3, the SNR and the CNR increase by about 10%. The improvements in image quality make it possible to distinguish tiny low-contrast abnormal tissues with the same imaging doses. On the other hand, it has the potential to reduce the radiation doses under the same image quality.

## 4. Conclusions

As a conclusion, the few-view prereconstruction based tube current modulation strategy is proposed in this work. It is derived from the SNR analysis in the sinogram domain. The main idea behind the strategy is to make a more uniform distribution of the sinogram to enhance the CT image quality. In the strategy, exact allocation of the X-ray photon at various projection views is made with the reference sinogram provided by the prereconstruction of FRESH method. Its feasibility and effectiveness have been demonstrated by the experiment results. The SNR and CNR of the final reconstruction are enhanced by more than 10% using the proposed method. In general, a lowered imaging dose results in a high noise level of CT image. But the noise can be reduced if the proposed strategy is adopted. Therefore the proposed strategy has the potential to achieve the same noise levels of CT image while reducing the overall radiation dose to patients. We will apply it to the practical CT system and make more experiments for the quantitative analysis in the future.

## Figures and Tables

**Table 1:** The geometrical configurations in the numerical simulations.

Scanning configuration parameters	Values
Trajectory radius (cm)	40.0
Object radius (cm)	22.5
Source-to-detector distance (cm)	70.0
Projection number per circle	1024
Linear array detector size (cm)	76.8
Detector unit number	512
Reconstructed image dimensions	512 × 512

**Table 2:** The SNR and CNR of the reconstructed images in Figure 4.

Reconstructed images	Figure 4(a)	Figure 4(b)	Figure 4(c)
SNR			
R1	10.93	11.55	15.71
R2	17.26	20.01	24.63
R3	15.93	21.73	30.94
R4	23.14	28.00	27.29
CNR			
R2 and R3	1.58	1.81	2.58

**Table 3:** The SNR and CNR of the reconstructed images in Figure 6.

Reconstructed images	Figure 6(a)	Figure 6(b)	Figure 6(c)
SNR			
R1	6.54	7.28	8.59
R2	6.34	6.00	7.11
R3	12.62	10.88	13.43
R4	13.18	14.29	14.41
CNR			
R5 and R6	1.01	1.02	1.12
